# Antioxidant Barrier, Redox Status, and Oxidative Damage to Biomolecules in Patients with Colorectal Cancer. Can Malondialdehyde and Catalase Be Markers of Colorectal Cancer Advancement?

**DOI:** 10.3390/biom9100637

**Published:** 2019-10-22

**Authors:** Justyna Zińczuk, Mateusz Maciejczyk, Konrad Zaręba, Wioletta Romaniuk, Adam Markowski, Bogusław Kędra, Anna Zalewska, Anna Pryczynicz, Joanna Matowicka-Karna, Katarzyna Guzińska-Ustymowicz

**Affiliations:** 1 Department of Clinical Laboratory Diagnostics, Medical University of Bialystok, Waszyngtona 15a, 15-269 Białystok, Poland; matowic@umb.edu.pl; 2 Department of Hygiene, Epidemiology and Ergonomics, Medical University of Bialystok, Mickiewicza 2c, 15-222 Białystok, Poland; 3 2nd Clinical Department of General and Gastroenterological Surgery, Medical University of Bialystok, M. Skłodowskiej-Curie 24a, 15-276 Białystok, Poland; konrad.zareba@umb.edu.pl (K.Z.); chgastro@umb.edu.pl (B.K.); 4 Department of Haematology, Medical University of Bialystok, M. Skłodowskiej-Curie 24A, 15-276 Białystok, Poland; wioletta.romaniuk@vp.pl; 5 Department of Internal Medicine and Gastroenterology, Polish Red Cross Memorial Municipal Hospital, Sienkiewicza 79, 15-003 Bialystok, Poland; markowski@szpitalpck.pl; 6 Department of Conservative Dentistry, Medical University of Bialystok, M. Skłodowskiej-Curie 24A, 15-276 Białystok, Poland; azalewska426@gmail.com; 7 Department of General Pathomorphology, Medical University of Bialystok, Waszyngtona 13, 15-269 Białystok, Poland; pryczynicz.anna@gmail.com (A.P.); kguzinska74@gmail.com (K.G.-U.)

**Keywords:** colorectal cancer, redox biomarkers, oxidative stress, antioxidants

## Abstract

This study is the first to assess the diagnostic utility of redox biomarkers in patients with colorectal cancer (CRC). Antioxidant barrier (Cu,Zn-superoxide dismutase (SOD), catalase (CAT), glutathione peroxidase (GPx), glutathione reductase (GR), uric acid (UA), reduced glutathione (GSH)), redox status (total antioxidant (TAC)/oxidant status (TOS), ferric reducing ability (FRAP)), and oxidative damage products (advanced glycation end products (AGE), advanced oxidation protein products (AOPP), malondialdehyde (MDA)) were measured in serum/plasma samples of 50 CRC patients. The activity of SOD was significantly higher whereas the activity of CAT, GPx and GR was considerably lower in CRC patients compared to the control group (*p* < 0.0001). Levels of UA, TOS, and OSI and concentrations of AGE, AOPP, and MDA were significantly higher, and the levels of GSH, TAC, and FRAP were considerably lower in CRC patients compared to the healthy controls (*p* < 0.0001). AUC for CAT with respect to presence of lymph node metastasis was 0.7450 (*p* = 0.0036), whereas AUC for MDA according to the depth of tumour invasion was 0.7457 (*p* = 0.0118). CRC is associated with enzymatic/non-enzymatic redox imbalance as well as increased oxidative damage to proteins and lipids. Redox biomarkers can be potential diagnostic indicators of CRC advancement.

## 1. Introduction

Colorectal cancer (CRC) is one of the most common types of cancer and classified as the third most common cancer in the world. According to GLOBOCAN estimates, in 2020 the number of new cases of colorectal cancer will amount to over 1.8 million, and the number of deaths will come to 10,502,507 [1]. It is important to detect cancer at an early stage because the five-year relative survival rate for patients with stage I colorectal cancer is about 92%, whereas in patients with stage IV (with lymph node and distant metastases) is less than 10%. However, despite screening tests, i.e., faecal occult blood testing (FOBT) and colonoscopy (‘gold standard’ of CRC screening), the survival time of patients with colorectal cancer has not improved significantly [2]. The main cause of this fact is that colonoscopy is an invasive and limited method due to difficulties for patients before the test performance. On the other hand, faecal occult blood testing offers limited sensitivity, particularly at the initial stage of the disease [2]. Nevertheless, early detection and treatment of CRC may prevent further progression of invasive cancer as well as significantly reduce the percentage of patient mortality. Therefore, it is important to understand the biology of CRC and search for new diagnostic biomarkers enabling the detection of cancer at its early stage.

Colorectal cancer is a consequence of multiple genetic events. Nevertheless, the majority of CRC cases occur as a result of the CIN (chromosomal instability) pathway characterized by defects in chromosomal segregation, telomere stability, and DNA damage response [3]. One of the most important mechanisms responsible for DNA damage is oxidative stress [4]. This process is defined as an imbalance between the production of reactive oxygen (ROS) and nitrogen (RNS) species and the efficiency of enzymatic and non-enzymatic antioxidant protection [5]. Overproduction of ROS is a result of the exposure to risk factors including smoking, stress, alcohol, toxins and inflammatory process caused by metabolic diseases, as well as lifestyle factors, diet, and dysbiosis, which are also connected with the development of colorectal cancer [6,7]. Increase of ROS level may lead to redox imbalance and cause tumour initiation and progression by activation of redox-responsive signalling cascades that promote cell growth [8]. Moreover, ROS induce lipid and protein peroxidation. It is well known that oxidized proteins accumulate in cells and inhibit proteasome activity, thus increasing the accumulation of misfolded/damaged proteins and causing further structural and functional alterations of cell organelles [9]. It has been confirmed that oxidative stress is involved in the development of malignant tumours through genetic mutations and initiation of DNA damage; inhibition of apoptosis; as well as promotion of proliferation, differentiation, and migration of malignant cells [10]. The usefulness of oxidative stress biomarkers has been demonstrated in numerous systemic diseases such as obesity, insulin resistance, diabetes, chronic kidney disease, and dementia, as well as in some types of cancer such as gastric cancer, ovarian cancer, and melanoma [7,11,12,13,14,15]. However, still little is known about the diagnostic utility of oxidative stress/redox parameters in patients with colorectal cancer. No study has also measured the total antioxidant/oxidant capacity in CRC patients. Therefore, the aim of our study was to evaluate the redox status, enzymatic and non-enzymatic antioxidants, as well as oxidative damage to proteins and lipids in colorectal cancer patients compared to the healthy controls. We are also the first to assess the diagnostic utility of oxidative stress parameters using the analysis of receiver operating characteristic (ROC) curve.

## 2. Materials and Methods

### 2.1. Patients

The research was approved by the Bioethics Committee of the Medical University of Bialystok, Poland (permission number R-I-002/48/2019). After a thorough explanation of the purpose of the study and possible risks, all the qualified patients consented in writing to participate in the experiment. The study was conducted in accordance with the World Medical Association Declaration of Helsinki for ethical principles for medical research involving human subjects.

The study group consisted of 50 patients (19 women and 31 men) treated surgically for colorectal cancer in the 2nd Clinical Department of General and Gastroenterological Surgery at the Medical University of Bialystok Clinical Hospital in the years 2017–2018. Patients were selected based on the following criteria: patients of both sexes without coexistence of other systemic diseases who had not been treated by radio- or chemotherapy before the surgery. The time from diagnosing a patient with cancer to the surgery varied from a minimum of two days to a maximum of four weeks. The study material was collected from all patients before surgical resection of tumour. Differentiation and recognition of histological type of cancer were performed following the World Health Organization guidelines [16]. Tumour stage was determined according to the TNM classification standard of the Union for International Cancer Control. TNM classification of malignant tumours included pT (depth of invasion), pN (lymph node metastasis), and pM (distant metastasis) stages [17]. 

In the control group, selected by sex and age to match the study group, samples were obtained from 40 healthy subjects attending follow-up visits at the Specialist Dental Clinic (Department of Restorative Dentistry) at the Medical University of Bialystok from January 2018 to January 2019. Only patients with normal results of complete blood count and biochemical blood tests (Na^+^, K^+^, creatinine, INR, ALAT, ASPAT) were admitted to the control group.

The exclusion criterion in patients with CRC and the control group was any systemic or autoimmune disease (diabetes, insulin resistance, hypertension, coronary heart disease, rheumatoid arthritis, and psoriasis) as well as thyroid, lung, kidney, liver, gastrointestinal, and infectious diseases (HCV and HIV infection) as well as immunity disorders. Additionally, smokers and patients taking antibiotics, glucocorticosteroids, non-steroidal anti-inflammatory drugs (NSAIDs), vitamins, and dietary supplements for last 3 months were excluded from the study and the control groups. In addition, only subjects on a standard diet were qualified for the study (2000 calories; 55% carbohydrates, 30% fat, and 15% protein).

The number of patients in the study and the control group was set based on a previously conducted pilot study. The power of the study was set at 0.9.

### 2.2. Blood Collection

Fasting venous blood (10 mL) was collected from all patients on empty stomach and upon overnight rest. The S-Monovette® K3 EDTA blood collection system (Sarstedt, Germany) was used for this. Blood was centrifuged at 1500× *g* for 10 min at +4 °C (MPW 351, MPW Med. Instruments, Warsaw, Poland) and the top layer (plasma) was taken. In order to prevent sample oxidation, 0.5 M butylated hydroxytoluene (20 μL/2 mL plasma or serum) was added [18]. Until redox determinations, all samples were stored at −80 °C.

### 2.3. Determination of Redox Markers

All the assays were performed in duplicate samples. The activity of enzymatic antioxidants was analysed in the serum samples, while the total antioxidant/oxidant status as well as concentrations of non-enzymatic antioxidants and oxidative damage products—in the plasma samples. The absorbance/florescence was measured using Infinite M200 PRO Multimode Microplate Reader (Tecan). The results were standardized to 100 mg of total protein. The content of total protein was estimated colorimetrically at 562 nm wavelength via the bicinchoninic acid (BCA) method [19]. A commercial kit was used (Thermo Scientific PIERCE BCA Protein Assay; Rockford, IL, USA).

### 2.4. Enzymatic and Non-Enzymatic Antioxidants 

The activity of copper-zinc-superoxide dismutase (SOD, E.C. 1.15.1.1) was analysed colorimetrically at 480 nm wavelength by measuring the inhibition of adrenaline oxidation to adrenochrome [20]. One unit of SOD activity was defined as the amount of enzyme inhibiting adrenaline oxidation by 50%. The activity of catalase (CAT, E.C. 1.11.1.6) was estimated colorimetrically at 240 nm wavelength by measuring the rate of hydrogen peroxide decomposition [21]. One unit of CAT activity was defined as the amount of enzyme that decomposes 1 mmol of hydrogen peroxide per 1 min. The activity of glutathione peroxidase (GPx, E.C. 1.11.1.9) was analysed colorimetrically at 340 nm wavelength based on the reduction of organic peroxides in the presence of reduced nicotinamide adenine dinucleotide phosphate (NADPH) [22]. One unit of GPx activity was defined as the amount of enzyme catalysing the oxidation of 1 μmol of NADPH per 1 min. The activity of glutathione reductase (GR, E.C. 1.8.1.7) was determined colorimetrically at 340 nm wavelength by measuring the decrease in NADPH absorbance [23]. One unit of GR activity was defined as the quantity of enzyme that catalyses the oxidation of 1 μmol of NADPH per 1 min.

The level of uric acid (UA) was estimated colorimetrically at 590 nm wavelength. Commercial kit from BioAssay Systems, Harward, CA, USA (QuantiChromTM Uric Acid DIUA-250 kit) was used. The level of reduced glutathione (GSH) was measured colorimetrically based on the reaction of GSH from the sample with 5,5′-dithiobis-2-nitrobenzoic acid (DTNB) [24]. The absorbance of the resulting complex was measured at 412 nm wavelength.

### 2.5. Total Antioxidant/Oxidant Status

The total antioxidant capacity/status (TAC) was analysed colorimetrically based on the reaction of ABTS (2,2-azinobis-3-ethylbenzothiazoline-6-sulfonic acid) radical cation with antioxidants contained in the plasma [25]. TAC levels were calculated from the calibration curve for 6-hydroxy-2,5,7,8-tetramethylchroman-2-carboxylic acid (Trolox). The total oxidant status (TOS) was measured bichromatically (560/800 nm) based on the oxidation of Fe^2+^ to Fe^3+^ in the presence of the oxidants contained in the plasma [26]. TOS levels were expressed as micromolar hydrogen peroxide equivalent per litre. Oxidative stress index (OSI) was calculated by dividing TOS level by TAC level (TOS/TAC ratio) and expressed in % [27]. 

Ferric reducing ability of plasma (FRAP) was analysed colorimetrically at 593 nm wavelength using 2,4,6-tripyridyl-s-triazine (2,4,6-TPTZ) [28]. FRAP levels were calculated from the calibration curve for FeSO_4_.

### 2.6. Oxidative Damage Products

The content of advanced glycation end products (AGE) was estimated fluorimetrically by measuring AGE-specific fluorescence at 350 nm/440 nm wavelength in 96-well black bottom microplates [29]. For AGE determination, plasma samples were diluted 1:50 (*v*/*v*) in phosphate buffered saline (0.02 M, pH 7.0). The concentration of advanced oxidation protein products (AOPP) was analysed colorimetrically at 340 nm wavelength by measuring the oxidative capacity of iodine ion [29]. For AOPP determination, plasma samples were diluted 1:50 (*v*/*v*) in in phosphate buffered saline (0.02 M, pH 7.0).

The concentration of malondialdehyde (MDA) was determined colorimetrically at 535 nm using the thiobarbituric acid reactive substances (TBARS) method [30]. 1,3,3,3 tetraethoxypropane was used as a standard.

### 2.7. H + E Staining

Histopathological diagnosis (histological type, the grade of histological malignancy, as well as clinical-anatomical advancement) has been established based on haematoxylin-eosin (H + E) staining. Each tumour was cut along a line that was parallel to the longest tumour axis. In this way, 4 to 8 slices contained cancer and adjacent macroscopically unchanged tissues of 1–1.5 cm in size were taken. Tissues were fixed in 10% buffered formalin within 24 to 48 hours. The specimens were embedded in paraffin at a temperature of 56 °C. Paraffin blocks were cut into 4 μm thick sections. The obtained sections were stained with H + E and reviewed by two independent pathologists on a microscope Olympus CX22 (200× or 400× magnification).

### 2.8. Statistical Analysis

Statistical analysis was performed using the Statistica 10.0 system (StatSoft, Krakow, Poland) and GraphPad Prism 7.0 (GraphPad Software). The Shapiro-Wilk test was used to examine normal distribution. For normal distribution of the results, a Student’s t-test was used. In the lack of normal distribution of the results, the Mann–Whitney U test was used. The correlations between the measured parameters were analysed by Pearson correlation coefficient. Statistical significance was established at *p* < 0.05. The diagnostic value of oxidative stress parameters and the optimum cut-off values were determined based on receiver operating characteristic (ROC) analysis.

## 3. Results

### 3.1. Clinical Findings

**Figure 1:**
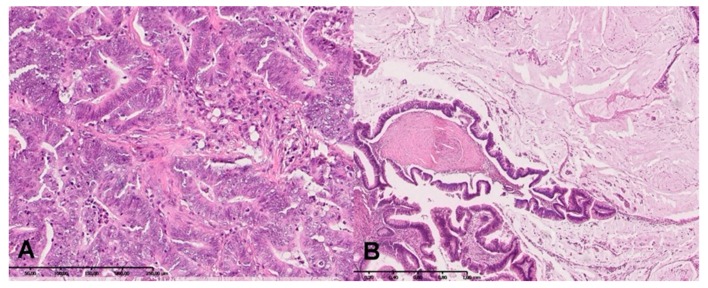
(**A**) Colorectal adenocarcinoma without mucinous component. (**B**) Adenocarcinoma with mucinous component. H+E staining.

The study included 50 patients with colorectal cancer and 40 healthy people matched by age and sex with the study group. In all CRC patients the tumour differentiation grade was G2 (moderately differentiated). A total of 40 patients had adenocarcinoma and 10 patients had a mucinous adenocarcinoma (Figure 1A,B). Differentiation and recognition of histological type of cancer were performed following the World Health Organization guidelines [16], while tumour stage was determined according to the TNM classification standard of the Union for International Cancer Control. There was no significant relationship between age and sex of patients with clinical and demographic parameters. Detailed patient characteristics are summarized in Table 1.

### 3.2. Antioxidant Defence

**Figure 2:**
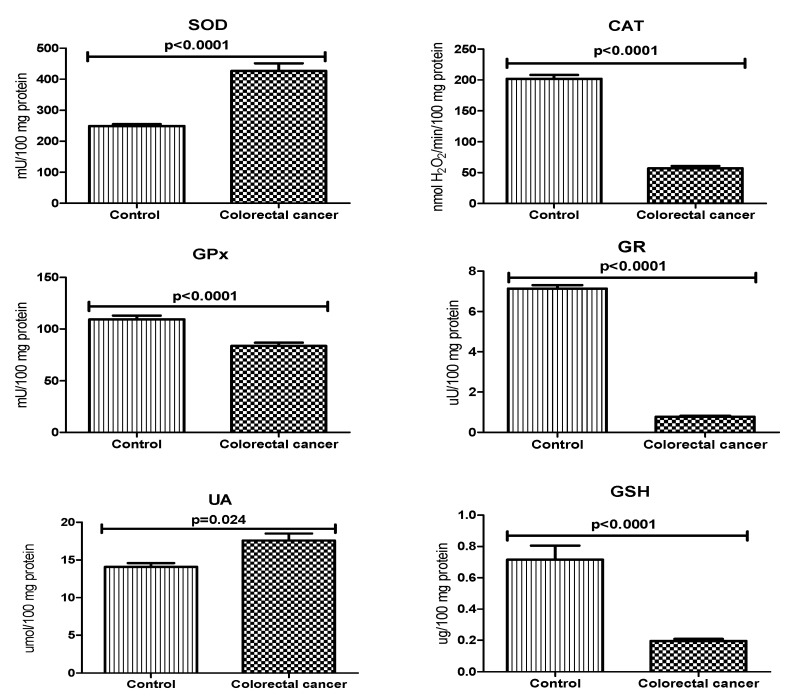
Non-enzymatic and enzymatic antioxidant defence in patients with colorectal cancer and the control group. Abbreviations: SOD, superoxide dismutase-1; CAT, catalase; GPx, glutathione peroxidase; GR, glutathione reductase; UA, uric acid; GSH, reduced glutathione.

In order to assess the antioxidant barrier, we evaluated the activity of antioxidant enzymes (i.e., Cu,Zn-superoxide dismutase (SOD), catalase (CAT), glutathione peroxidase (GPx), and glutathione reductase (GR)) and concentration of non-enzymatic antioxidants (uric acid (UA), and reduced glutathione (GSH)). SOD activity was significantly higher, whereas CAT activity was considerably lower in the serum of patients with colorectal cancer compared to the control group (*p* < 0.0001, *p* < 0.0001, respectively). The concentration of UA in plasma was significantly higher in the study group than in the controls (*p* < 0.05). There was a statistically significant increase in the activity of GPx, GR and GSH in colorectal cancer patients compared to healthy controls (*p* < 0.0001, *p* < 0.0001, *p* < 0.0001) (Figure 2).

### 3.3. Total Antioxidant/Oxidant Status

**Figure 3:**
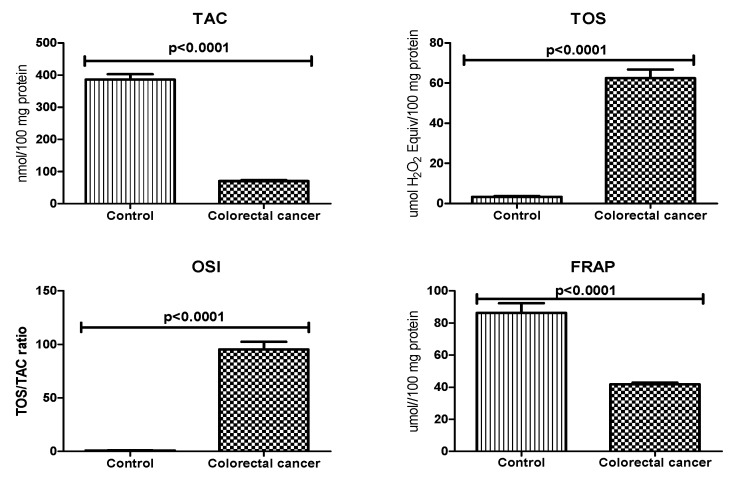
Total antioxidant/oxidant status in patients with colorectal cancer and the control group. Abbreviations: TAC, total antioxidant capacity; TOS, total oxidant status; OSI, oxidative stress index; FRAP, ferric reducing ability of sample.

To assess the redox status, we focused on total antioxidant capacity (TAC), total oxidant status (TOS), and ferric reducing ability of plasma (FRAP). Furthermore, we also calculated oxidative stress index (OSI) by dividing TOS level by TAC level (TOS/TAC ratio). In the plasma of patients with colorectal cancer, TAC and FRAP levels were significantly lower compared to the control group (*p* < 0.0001). We observed considerable increase in TOS in the plasma of study group patients compared to healthy controls (*p* < 0.0001). The OSI value in colorectal cancer patients was significantly higher than in the healthy controls (*p* < 0.0001) (Figure 3).

### 3.4. Oxidative Damage Products

**Figure 4:**
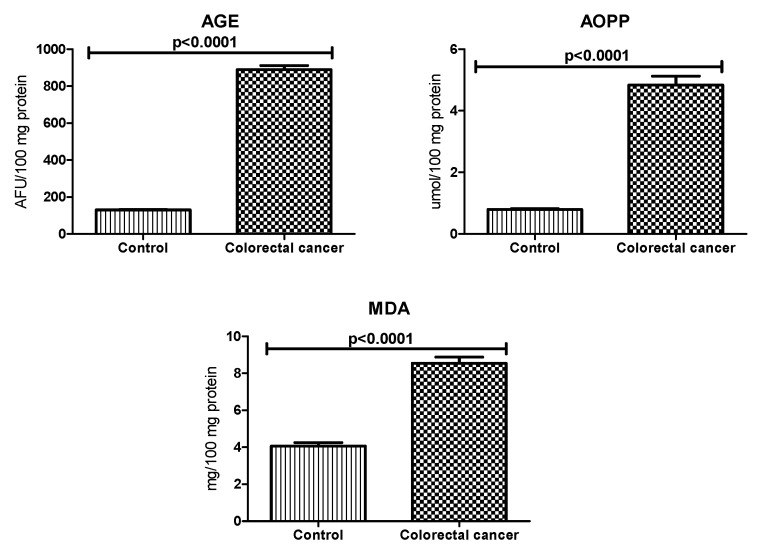
Oxidative damage to proteins and lipids in patients with colorectal cancer and the control group. Abbreviations: AGE, advanced glycation end products; AOPP, advanced oxidation protein products; MDA, malondialdehyde.

To assess the oxidative stress, we used oxidative damage products of proteins (i.e., advanced glycation end products (AGE), advanced oxidation protein products (AOPP)) and lipids (malondialdehyde (MDA)). There was a statistically significant increase in AGE fluorescence in plasma of colorectal cancer patients compared to AGE fluorescence in the control group (*p* < 0.0001). AOPP and MDA concentrations were also considerably higher in the plasma of the study group than in the control group (*p* < 0.0001, *p* < 0.0001) (Figure 4).

### 3.5. ROC Analysis

**Figure 5:**
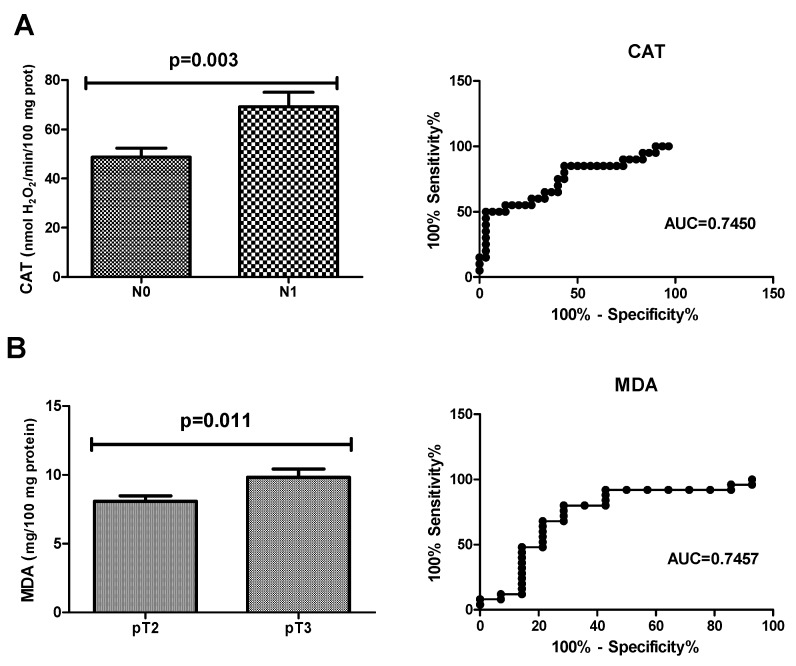
(**A**) ROC analysis of CAT in CRC patients with (N1 + N2) and without (N0) regional lymph node metastasis. (**B**) ROC analysis of MDA in CRC patients with the tumour grown into the muscularis propria (pT2) and with the tumour grown through the muscularis propria and into the subserosa (pT3). Abbreviations: AUC, area under curve; ROC, receiver operating characteristic.

ROC analysis was performed in the study to assess the diagnostic value of oxidative stress biomarkers in the diagnostics of colorectal cancer (Table 2, Table 3 and Table 4). We demonstrated that all determined redox parameters differentiate CRC patients from healthy controls to a large extent. We also proved that oxidative stress biomarkers are useful in the differential diagnosis of CRC patients. Particular attention should be paid to CAT for which AUC in the presence of lymph node metastasis was 0.7450 with cut-off value >61.61 nmol H_2_O_2_/min/100 mg protein, 65.00% sensitivity and 66.67% specificity (Table 3, Figure 4). We also showed a very high diagnostic value of MDA determination (AUC 0.7457, *p* = 0.0118) in differentiating the group of patients with CRC at stage pT2 of tumour invasion from patients with stage pT3 CRC. The cut-off value was <9.361 mg/100 mg protein with sensitivity of 72.00% and specificity of 71.43% (Table 4, Figure 5).

### 3.6. Correlations

The results of all statistically significant correlations are presented in Table 5. Interestingly, we observed a positive correlation between MDA and CEA level as well as between MDA and depth of tumour invasion (pT) in CRC patients. Interestingly, the concentration of MDA remarkably positively correlated with CRP levels. We also demonstrated a negative correlation between AGE and UA and the number of eosinophils as well as between GPx and CEA. Moreover, there were also statistically negative correlations between AGE and neutrophils, SOD and monocytes, and TOS and basophiles.

## 4. Discussion

Our study is the first to assess the diagnostic utility of redox biomarkers in patients with colorectal cancer. We have demonstrated disturbances in enzymatic and non-enzymatic antioxidants as well as enhanced oxidation of proteins and lipids in serum/plasma of CRC subjects. Moreover, we assume that catalase and malondialdehyde—a product of lipid peroxidation—may be potential non-invasive biomarkers differentiating tumour invasion depth or indicating the occurrence of lymph node metastasis.

One of the most important mechanisms associated with carcinogenesis is oxidative stress. This phenomenon is defined as alterations in gene expression, cell metabolism, and cell homeostasis caused by overproduction of ROS and disturbances in antioxidant mechanisms [31]. Antioxidant enzymes form our first line of defence against oxidative damage. A critical ROS-scavenging enzyme is superoxide dismutase (SOD) which converts superoxide anion to hydrogen peroxide and molecular oxygen. In our study, SOD activity was significantly higher in patients with colorectal cancer, which suggests an adaptive response to increased formation of ROS and RNS. Similarly, in patients with CRC, we observed a considerably higher concentration of uric acid, the most important plasma non-enzymatic antioxidant. It is well known that strengthening the antioxidant barrier is the basic mechanism protecting the body against enhanced production of free radicals and associated oxidative stress. Indeed, in CRC subjects, ROS overproduction occurs through alterations in mitochondrial function/energy metabolism as well as enzyme inactivation during the tumourigenesis [32]. However, the activity/concentration of other enzymatic (CAT, GR, GPx) and non-enzymatic (GSH) antioxidants was significantly lower in patients with CRC compared to healthy people. Therefore, antioxidant reserves could be exhausted in CRC subjects, which predisposes them to redox imbalance as well as oxidative damage. It has been demonstrated that colon tumour tissue produces particularly large amounts of hydrogen peroxide (H_2_O_2_) [33]. Although H_2_O_2_ is not a free radical, it can pass through the cell membrane and—in the presence of transition metal ions (e.g. Cu^2+^ and Fe^2+^)—transform into a hydroxyl radical [10]. Hydroxyl radical has very high biological reactivity and is destroys macromolecules (proteins, lipids, DNA/RNA) as well as cell structures. However, H_2_O_2_ can also react with superoxide anion in Haber-Weiss reaction, which further boosts the formation of hydroxyl radicals [8,34]. Therefore, it is not surprising that we have observed decreased activity of GPx and CAT, the enzymes responsible for the elimination of hydrogen peroxide. Moreover, it can be assumed that free radicals, such as superoxide anion, hydroxyl, alkoxyl, and peroxyl radicals, could inactivate antioxidant enzymes through its structural modification and finally lead to decreasing the activity of CAT, GPx, and GR [35].

Enzymatic and non-enzymatic antioxidants closely interact with each other. Therefore, it is difficult to deduce the redox status based on the evaluation of individual antioxidants alone. Consequently, we were the first to evaluate the biomarkers characterizing the resultant antioxidant capacity (TAC, FRAP, TOS, OSI) in colorectal cancer patients. Indeed, TAC and FRAP reflect the total content of antioxidants, while TOS is the sum of all oxidants contained in the sample [25,26,28]. In our work, we found lower TAC and FRAP levels, which indicates weakened antioxidant barrier resulting from excessive free radical production (↑TOS in CRC patients). Moreover, oxidative stress index (OSI) was significantly higher in colorectal cancer patients. It is well known that OSI provides more information about the interactions between oxidants and ROS-scavengers [34]. Therefore, enhanced OSI level in CRC patients suggests that oxidation processes outweigh the antioxidant protection. Due to the shift of redox balance to the oxidation side, patients with CRC are particularly sensitive to oxidative stress and oxidative damage. Indeed, the colon intestinal mucosa is constantly exposed to pro-oxidative and carcinogenic factors derived from the diet as well as bacteria [36]. Human gut microflora contains up to 500 bacteria species that can generate ROS and RNS [37]. Moreover, a high-fat/high-carbohydrate diet can induce systemic oxidative stress [38]. It is believed that inflammation and oxidative stress associated with metabolic disturbances constitute an environment conducive to the development of CRC [39]. Thus, it is a not surprising increase in the CRC incidence in patients with obesity, insulin resistance as well as metabolic syndrome [40,41]. 

ROS overproduction increases with cancer progression and results in lipid peroxidation and protein oxidative damage [42]. Malondialdehyde (MDA)—the final product of lipoperoxidation—is a highly electrophilic molecule that reacts with cell nucleophiles to form MDA adducts and MDA oligomers. Furthermore, malondialdehyde reacts with several nucleic acids forming deoxyguanosine (dG), deoxyadenosine (dA), and deoxycytidine (dC) adducts [43]. It was shown that MDA–DNA oxidation products have pro-mutagenic properties and induce mutations in oncogenes/tumour suppressor genes in human tumours [44]. In our study, MDA level was significantly higher in colorectal cancer patients compared to the healthy controls. Among the many lipid peroxidation products, such as MDA, 4-hydroxy-2-nonenal (4-HNE), 4-hydroxyhexenal (4-HHE), and 8-isoprostanes (8-isoP), MDA is the one with the highest mutagenicity and carcinogenicity [43]. However, increased formation of ROS/RNS in CRC patients also leads to enhanced oxidative damage to proteins and polypeptides. Protein oxidation can lead to ROS-induced modifications of amino acid residues/prosthetic groups of enzymes, as well as fragmentation/aggregation of proteins in the cell [45]. The most frequently evaluated biomarkers of oxidative protein damage are advanced oxidation protein products (AOPPs) and advanced glycation end products (AGEs). AOPPs are dityrosine containing crosslinked protein products. It was demonstrated that AOPPs trigger oxidative ignition of monocytes, neutrophils, and T lymphocytes, resulting in a highly up regulation of dendritic cells [46]. Under these conditions, the NfkB signalling pathway is also activated, which not only increases the production of pro-inflammatory cytokines, but also enhances free radical formation [31]. AGEs have a very similar structure to AOPPs. Therefore, both AGEs and AOPPs stimulate proinflammatory response (by interacting with receptor for advanced glycation end products, RAGE) and induce transendothelial chemotaxis of human monocytes [31]. Therefore, AGEs and AOPPs enhance the release of tumour necrosis factor α (TNF-α), interleukin-1 (IL-1) and interleukin-6 (IL-6). AGEs can also increase thrombogenicity and permeability of endothelial cells, i.e., factors that mark the participation of advanced glycation end products in cancer proliferation and/or cancer progression [46]. In this study, all the assessed protein modification markers (AGE, AOPP) were significantly higher in colorectal cancer patients as compared to the control group. To date, no data was found about AOPP and AGE level in plasma/serum samples of colorectal cancer patients. However, increased oxidative protein damage has been observed in gastric cancer [47], thyroid cancer [48], breast cancer [49], and oral squamous cell carcinoma [50], which correlates with cancer progression and tumour metastasis. The fact is not surprising as oxidatively modified proteins tend to form aggregates resistant to degradation by proteolytic enzymes. This promotes the accumulation of altered proteins in cells and leads to a gradual loss of their structure and biological function [34]. Indeed, disturbances in protein breakdown processes are involved in the development of progressive cancer [47]. Therefore, should antioxidants be used in patients with CRC? Recent studies indicate that supplementation with antioxidants can both inhibit as well as intensify tumour initiation/progression [51,52,53,54]. Interestingly, as demonstrated in the epidemiological studies, an increasing amount of antioxidants in the diet generally does not improve the prognosis of cancer patients [51,53]. In addition, it has been proven that oxidative stress increases in metastasizing cells as well as reduces distant metastasis, which opens new possibilities in the treatment of colorectal cancer patients [51]. However, there is a need for further research in this area in both the in vitro model as well as clinical trials.

Despite the available diagnostic methods for colorectal cancer detection, an ideal non-invasive biomarker of CRC has not been discovered yet [55,56]. The most commonly evaluated laboratory indicator of CRC is carcinoembryonic antigen (CEA) discovered over 50 years ago [57]. Although CEA increased in about 60% to 85% colorectal cancer patients, its specificity was 90% but sensitivity only 40 to 75%. Moreover, the content of carcinoembryonic antigen rarely increased in stage I CRC and appeared to be unable to differentiate benign lesions with malignant polyps [57]. Therefore, carcinoembryonic antigen cannot be used as a standard biomarker in colorectal cancer diagnosis and is not recommended for screening tests. This is also confirmed by the results of our study, in which CEA remained within reference values range: 72% of CRC patients. Thus, further development of non-invasive diagnostic methods is essential as it would enable early diagnosis at pre- and postoperative stages and offer a wider selection of most suitable therapeutic methods and post-treatment follow-up for a given patient.

Considering that oxidative stress is the cause of numerous human diseases, redox biomarkers are becoming increasingly common in the clinical practice. The diagnostic usefulness of redox indicators has been demonstrated in genetic diseases (e.g. Down syndrome, ataxia-telangiectasia) [58,59], metabolic disorders (obesity, insulin resistance, type 1 and 2 diabetes) [12,60], inflammatory diseases (chronic kidney disease and non-alcoholic fatty liver disease) [13,61], as well as in some types of cancer, such as gastric cancer, ovarian cancer, and melanoma. In the present study, we were the first to demonstrate the usefulness of redox biomarkers in the diagnosis and/or assessment of CRC severity. We observed that all of the determined redox parameters significantly distinguish CRC patients from the healthy controls (with very high sensitivity and specificity). Furthermore, we also demonstrated the usefulness of redox biomarkers in differential diagnosis of CRC patients. Indeed, ROC analysis showed that CAT clearly indicates the occurrence of lymph node metastasis or its lack in these patients. Also noteworthy is the positive correlation between CAT activity and CA 19-9 level. It is well known that cancer features such as nodal status (pN) and depth of primary tumour infiltration (pT) are the most important prognostic factors for local recurrence and distant metastasis in cancer patients [62]. However, the only reliable test to assess and confirm the cancer stage is the histopathological analysis of resected tumour and the surrounding tissues. Therefore, the discovery of new biomarkers to be assayed in serum/plasma samples may be helpful in pre-operative diagnosis and would enable non-invasive evaluation of cancer advancement, thereby facilitating the choice of appropriate treatment, and improvement of CRC patients’ survival rate. Additionally, we demonstrated a very high diagnostic value of MDA determination in colorectal cancer diagnosis. In a group of CRC patients, the evaluation of plasma MDA allows to distinguish those with depth of tumour invasion at stage pT2 from patients with stage pT3 (AUC 0.7133, *p* = 0.037). Thus, our results suggest that lipid peroxidation in CRC patients is enhanced with the increase of depth of tumour invasion. The differences between both groups may be associated with high cytotoxic potential of MDA that reveals intensified operation as a carcinogenic agent and promoter of CRC development and progression [47]. It is probable that MDA cytotoxicity increases along with cancer advancement, which may indicate that MDA can be engaged in cancer growth and connected with depth of colon wall infiltration. We concluded that MDA may be a useful marker in CRC advancement evaluation. Our observations are also confirmed by a positive correlation between MDA and CEA/CRP levels, as well as between MDA and depth of tumour invasion in CRC patients.

Finally, it is also worth considering certain limitations of our experiment. The evaluated redox biomarkers are not specific to colon cancer alone, so they can only be used after the elimination of other oxidative stress-related disorders. Additionally, the activity/concentration of antioxidants and the concentration of oxidation modification products were evaluated only in serum/plasma samples, which offers our results only an approximate value. However, the undoubted advantage of our work is a carefully selected group of CRC/control patients without any accompanying diseases as well as the fact that blood CAT and MDA can be used in non-invasive CRC diagnostics. Our experiment is also a starting point for further clinical trials assessing the diagnostic utility of redox biomarkers in a larger population of colorectal cancer patients.

## 5. Conclusions

Colorectal cancer is associated with enzymatic and non-enzymatic redox imbalance as well as increased oxidative damage to proteins and lipids as compared to healthy controls. Catalase and malondialdehyde can be potential non-invasive biomarkers indicating tumour invasion depth or presence of lymph node metastasis.

## Figures and Tables

**Table 1:** Characteristics of study group.

Parameter	n (%)
Age <60 >60	11 (22.0%) 39 (78.0%)
Sex male female	31 (62.0%) 19 (38.0%)
Histological type adenocarcinoma mucinous adenocarcinoma	40 (80.0%) 10 (20.0%)
Tumour location sigmoid colon rectum cecum ascending colon hepatic fold colon	15 (30.0%) 15 (30.0%) 7 (14.0%) 6 (12.0%) 5 (10.0%) 2 (4.0%)
Tumour’s size <3cm >3cm	15 (30.0%) 35 (70.0%)
pT—depth of invasion T1 T2 T3 T4	12 (24.0%) 14 (28.0%) 18 (36.0%) 6 (12.0%)
pN—lymph node metastasis N0 N1 N2	30 (60.0%) 12 (24.0%) 8 (16.0%)
pM—distant metastasis M0 M1	44 (88.0%) 6 (12.0%)
Stage at diagnosis I II III IV	11 (22.0%) 16 (32.0%) 18 (36.0%) 5 (10.0%)
CEA level (ng/mL) 0–5.0 >5.0	36 (72.0%) 14 (28.0%)

**Table 2:** Receiver operating characteristic (ROC) analysis of oxidative stress biomarkers of colorectal cancer patients and the controls. Abbreviations: SOD, superoxide dismutase-1; CAT, catalase; GPx, glutathione peroxidase; GR, glutathione reductase; UA, uric acid; GSH, reduced glutathione; TAC, total antioxidant capacity; TOS, total oxidant status; OSI, oxidative stress index; FRAP, ferric reducing ability of sample; AGE, advanced glycation end products; AOPP, advanced oxidation protein products; MDA, malondialdehyde.

Parameter	AUC	*p*-Value	Cut-Off	Sensitivity (%)	Specificity (%)	95% Confidence Interval
*Antioxidant defense*						
SOD (mU/100 mg protein)	0.9048	<0.0001	>279.7	87.80	90.48	0.8242–0.9854
CAT (nmol H_2_O_2_/min/100 mg protein)	0.9988	<0.0001	<123.3	97.56	97.50	0.9955–1.002
GPx (mU/100 mg protein)	0.9209	<0.0001	<99.54	90.24	93.10	0.8387–1.003
GR (uU/100 mg protein)	1.000	<0.0001	<4.205	100.0	100.0	1.000–1.000
UA (umol/100 mg protein)	0.6880	0.002335	>14.96	65.85	64.58	0.5756–0.8004
GSH (ug/100 mg protein)	0.9566	<0.0001	<0.2986	85.37	85.19	0.9124–1.001
*Redox status*						
TAC (nmol/100 mg protein)	1.000	<0.0001	<168.5	100.0	100.0	1.000–1.000
TOS (umol H_2_O_2_ Equiv/100 mg protein)	1.000	<0.0001	>14.27	100.0	100.0	1.000–1.000
OSI (TOS/TAC ratio)	1.000	<0.0001	>15.49	100.0	100.0	1.000–1.000
FRAP (umol/100 mg protein)	0.9377	<0.0001	<49.49	90.24	89.47	0.8726–1.003
*Protein and lipid oxidative damage*						
AGE (AFU/100 mg protein)	1.000	<0.0001	>322.0	100.0	100.0	1.000–1.000
AOPP (umol/100 mg protein)	1.000	<0.0001	>1.637	100.0	100.0	1.000–1.000
MDA (mg/100 mg protein)	0.9815	<0.0001	>5.669	92.68	92.00	0.9561–1.007

**Table 3:** Receiver operating characteristic (ROC) analysis of oxidative stress biomarkers of colorectal cancer patients with (N1+N2) and without lymph node metastasis (N0). Abbreviations: SOD, superoxide dismutase-1; CAT, catalase; GPx, glutathione peroxidase; GR, glutathione reductase; UA, uric acid; GSH, reduced glutathione; TAC, total antioxidant capacity; TOS, total oxidant status; OSI, oxidative stress index; FRAP, ferric reducing ability of sample; AGE, advanced glycation end products; AOPP, advanced oxidation protein products; MDA, malondialdehyde.

Parameter	AUC	*p*-Value	Cut-Off	Sensitivity (%)	Specificity (%)	95% Confidence Interval
*Antioxidant defense*						
SOD (mU/100 mg protein)	0.5507	0.6013	>426.9	53.33	52.17	0.3590–0.7425
CAT (nmol H_2_O_2_/min/100 mg protein)	0.7450	0.0036	>61.61	65.00	66.67	0.5989–0.8911
GPx (mU/100 mg protein)	0.6232	0.2044	>79.95	53.33	56.52	0.4421–0.8043
GR (uU/100 mg protein)	0.6435	0.1394	>0.7091	66.67	65.22	0.4677–0.8192
UA (umol/100 mg protein)	0.5681	0.4828	>16.49	53.33	52.17	0.3825–0.7537
GSH (ug/100 mg protein)	0.6029	0.2891	<0.1736	66.67	65.22	0.4185–0.7873
*Redox status*						
TAC (nmol/100 mg protein)	0.5318	0.7544	<70.33	46.15	47.83	0.3354–0.7282
TOS (umol H_2_O_2_ Equiv/100 mg protein)	0.5848	0.3864	<48.65	53.33	54.55	0.3931–0.7766
OSI (TOS/TAC ratio)	0.5245	0.8111	<85.56	53.85	54.55	0.3122–0.7368
FRAP (umol//100 mg protein)	0.5420	0.6650	<41.71	60.00	60.87	0.3512–0.7329
*Protein and lipid oxidative damage*						
AGE (AFU/100 mg protein)	0.6087	0.2628	<873.3	53.33	52.17	0.4204–0.7970
AOPP (umol/100 mg protein)	0.6290	0.1839	<4.388	66.67	65.22	0.4506–0.8074
MDA (mg/100 mg protein)	0.5188	0.8461	<8.707	60.00	60.87	0.3293–0.7084

**Table 4:** Receiver operating characteristic (ROC) analysis of oxidative stress biomarkers of colorectal cancer patients with the tumour grown into the muscularis propria (pT2) and with the tumour grown through the muscularis propria and into the subserosa (pT3). Abbreviations: SOD, superoxide dismutase-1; CAT, catalase; GPx, glutathione peroxidase; GR, glutathione reductase; UA, uric acid; GSH, reduced glutathione; TAC, total antioxidant capacity; TOS, total oxidant status; OSI, oxidative stress index; FRAP, ferric reducing ability of sample; AGE, advanced glycation end products; AOPP, advanced oxidation protein products; MDA, malondialdehyde.

Parameter	AUC	*p*-Value	Cut-Off	Sensitivity (%)	Specificity (%)	95% Confidence Interval
*Antioxidant defense*						
SOD (mU/100 mg protein)	0.5500	0.6265	>426.9	56.00	58.33	0.3361–0.7639
CAT (nmol H_2_O_2_/min/100 mg protein)	0.5633	0.5376	>49.17	56.00	58.33	0.3758–0.7509
GPx (mU/100 mg protein)	0.5600	0.5592	>79.22	48.00	50.00	0.3515–0.7685
GR (uU/100 mg protein)	0.6833	0.0744	>0.6881	68.00	66.67	0.4925–0.8742
UA (umol/100 mg protein)	0.6067	0.2992	>15.42	42.52	41.67	0.4228–0.7905
GSH (ug/100 mg protein)	0.5233	0.8203	>0.1857	44.00	41.67	0.3302–0.7164
*Redox status*						
TAC (nmol/100 mg protein)	0.5652	0.5316	>70.33	56.52	58.33	0.3342–0.7962
TOS (umol H_2_O_2_ Equiv/100 mg protein)	0.5818	0.4397	>47.87	56.00	54.55	0.3897–0.7739
OSI (TOS/TAC ratio)	0.5771	0.4729	>85.56	52.17	54.55	0.3704–0.7837
FRAP (umol//100 mg protein)	0.5033	0.9741	>43.19	40.00	41.67	0.2912–0.7154
*Protein and lipid oxidative damage*						
AGE (AFU/100 mg protein)	0.5133	0.8967	>873.3	48.00	50.00	0.3288–0.6978
AOPP (umol/100 mg protein)	0.5067	0.9483	<4.475	52.00	50.00	0.3054–0.7079
MDA (mg/100 mg protein)	0.7457	0.0118	<9.361	72.00	71.43	0.5678–0.9236

**Table 5:** Correlations of redox biomarkers in patients with colorectal cancer.

Pair of Variables	*r*	*p*
GPx and GR	0.575	<0.0001
FRAP and GSH	0.575	<0.0001
UA and AGE	0.407	0.008
CAT and AOPP	−0.341	0.029
GSH and AOPP	0.351	0.024
TOS and AOPP	0.650	<0.0001
OSI and AOPP	0.466	0.004
UA and CA 19-9	0.509	0.026
CAT and CA 19-9	0.642	<0.0001
GR and CA19-9	0.522	0.018
GPx and CEA	−0.448	0.036
MDA and CEA	0.560	0.008
UA and α1globulin	0.547	0.028
FRAP and location	0.332	0.045
MDA and pT	0.460	0.008
GPx and vascular invasion	0.512	0.043
MDA and CRP	0.980	<0.0001
FRAP and total cholesterol	0.670	0.009
UA and total cholesterol	0.565	0.035
UA and eosinophils	−0.663	0.037
SOD and monocytes	0.745	0.013
TOS and basophils	−0.735	0.016
AGE and eosinophils	−0.717	0.020
AGE and neutrophils	−0.636	0.047
